# Condomless anal intercourse among HIV-positive and HIV-negative men who have sex with men in Zimbabwe

**DOI:** 10.4102/sajhivmed.v25i1.1583

**Published:** 2024-09-30

**Authors:** Munyaradzi P. Mapingure, Innocent Chingombe, Tafadzwa Dzinamarira, Diego Cuadros, Grant Murewanhema, Brian Moyo, Chesterfield Samba, Amon Mpofu, Owen Mugurungi, Helena Herrera, Godfrey Musuka

**Affiliations:** 1 Innovative Public Health and Development Solutions, Harare, Zimbabwe; 2 ICAP, Columbia University, Harare, Zimbabwe; 3 Digital Epidemiology Laboratory, Digital Futures, University of Cincinnati, Cincinnati, United States of America; 4 Unit of Obstetrics and Gynecology, Faculty of Medicine and Health Sciences, University of Zimbabwe, Harare, Zimbabwe; 5 AIDS and TB Programmes, Ministry of Health and Child Care, Harare, Zimbabwe; 6 Gay and Lesbian Association of Zimbabwe (GALZ), Harare, Zimbabwe; 7 National AIDS Council, Harare, Zimbabwe; 8 School of Pharmacy and Biomedical Sciences, University of Portsmouth, Portsmouth, United Kingdom; 9 International Initiative for Impact Evaluation, Harare, Zimbabwe

**Keywords:** HIV, MSM, condomless anal intercourse, sexual behaviour, condom use, biobehavioural survey

## Abstract

**Background:**

Men who have sex with men (MSM) are disproportionately impacted by HIV in sub-Saharan Africa (SSA), where condomless anal intercourse (CAI) is a major driver of HIV transmission among this hidden subpopulation.

**Objectives:**

To determine CAI drivers and prevalence among HIV-positive and HIV-negative MSM.

**Method:**

Data from 1538 MSM who participated in a biobehavioural survey in Zimbabwe were used. Secondary statistical data analysis methods were used to determine prevalences and drivers of CAI.

**Results:**

A high prevalence of CAI, of at least 30%, among HIV-positive and HIV-negative MSM was found. Factors that led to a statistically significant higher CAI among HIV-positives compared to HIV-negatives included drunkenness (35% vs. 25%, *P* = 0.01), fear of partner (13% vs. 9%, *P* = 0.017), trusting the partner (10% vs. 6%, *P* = 0.008), and being offered more money (10% vs. 6%, *P* = 0.003).

**Conclusion:**

Our findings indicate that economic, socio-behavioural and perceptual dimensions increase men’s likelihood to engage in risky sexual behaviour, suggesting the need for HIV prevention efforts that provide tailored education regarding HIV risk among MSM in SSA. This is the first large biobehavioural survey that generated valuable information useful for analysing condomless anal sex among MSM in Zimbabwe.

**What this study adds:** This study adds to the body of knowledge on factors affecting use of condom during anal sex among MSM in Zimbabwe.

## Introduction

The global HIV epidemic among men who have sex with men (MSM) continues to be a significant public health concern worldwide, given the disproportionate burden of infection this group faces.^1^ Social stigma, criminalisation of homosexuality, and MSM-related policies that are either restrictive or absent have created a climate in which MSM populations have remained hidden, thus heightening the risk of acquiring and transmitting HIV to other MSM.^2^ These factors have also limited the feasibility of researching MSM, particularly in sub-Saharan Africa (SSA).^3^ Recently, research on MSM in SSA countries has found a high prevalence of HIV, ranging from 4% to 50%.^4^ The heightened risk among MSM of acquiring and transmitting HIV is primarily driven by condomless anal intercourse (CAI), one of the most efficient modes of HIV transmission.^5^ CAI is common among MSM in various African countries, with variations in associated risk factors. A biobehavioural survey (BBS) that used respondent-driven sampling (RDS) in Côte d’Ivoire found that 65% of MSM reported CAI.^6^ In Nigeria, the prevalence of CAI, defined as not using condoms at the most recent sex with a male partner, was 43%.^7^ In Cameroon, the prevalence of CAI, defined as CAI with a male partner in the last 6 months, was 57%.^8^

The primary drivers of CAI can be grouped into socio-behavioural, accessibility, and perceptual dimensions. Socio-behavioural drivers of CAI were perhaps the most multifaceted. Alcohol use, for example, which can impair judgement and decision-making, emerged as a significant factor. The influence of peer pressure or social norms, often leading to unpreparedness or embarrassment regarding procurement of condoms, is another crucial socio-behavioural factor. Furthermore, perceptions related to trust, where regular or attractive partners were less likely to use condoms, indicate that relationship dynamics also play a role in determining condom use. MSM might decide not to use condoms when having sex with a regular sexual partner due to the mutual trust that friends would not put them at risk.^9^ These dynamics may be influenced by misconceptions about HIV transmission risks, such as the erroneous belief that a partner not ejaculating inside reduces transmission risk. Likewise, condom unavailability could be a key determinant, which might be due to a lack of adequate distribution or logistical issues in specific areas, highlighting the need for improving access to condoms among this population. Furthermore, instances where MSM were offered more money to engage in CAI underscore the intersection of economic factors and sexual risk behaviour. Perceptual barriers, including disliking condoms or experiencing a rash from their use, further discourage condom usage. Likewise, the incorrect perception that anal sex is safe can also drive the prevalence of CAI.^6,7,8,9^

If the epidemic within the MSM population is not addressed, efforts to control HIV could be jeopardised, since the epidemic can spread to the entire population through the heterosexual practices of these men.^10^ A study in Côte d’Ivoire found factors associated with CAI to include a history of forced sex, alcohol consumption, having a regular partner and a casual partner, having bought sex, and self-perception of low HIV risk.^11^ In another study in Nigeria, prior HIV testing and knowledge of at least one sexually transmitted infection (STI) that can be transmitted through CAI were found to be protective against CAI.^5,12^

Therefore, there is a need to estimate the prevalence of CAI and its associated risk factors in diverse samples of MSM in a SSA context. As CAI is an essential modifiable proximal determinant of HIV transmission, a better understanding of factors associated with CAI can inform interventions to reduce HIV acquisition and transmission among this hidden high-risk subpopulation.

## Research methods and design

Between March 2019 and July 2019, ICAP at Columbia University, in collaboration with the Zimbabwe Ministry of Health and Child Care and the US Centers for Disease Control and Prevention (CDC), Zimbabwe, conducted a cross-sectional BBS with MSM and transgender women/gender queer (TGW/GQ) individuals in Harare and Bulawayo, Zimbabwe. The survey was conducted using the World Health Organization’s BBS Guidelines for Populations at Risk for HIV,^13^ and full details on the methods and primary outcomes have been published.^14,15^ Individuals were recruited using RDS, a chain referral approach used to recruit populations without a sampling frame. Individuals were eligible to participate if they were assigned male sex at birth; had engaged in oral or anal sex with a man in the past 12 months; were 18 years or older; had resided in Harare or Bulawayo for at least 1 month; spoke English, Shona, or Ndebele; and had provided written informed consent. The survey staff administered a structured questionnaire to all participants through a tablet device.^15^ The interviewer asked respondents ‘no’ and ‘yes’ questions about circumstances in which they tended to not use condoms. Open-ended questions that allowed respondents to mention other reasons were also used. All consenting participants were tested for HIV, regardless of their self-reported status, using an adapted version of the national three-test algorithm (Alere HIV Combo [Abbott, Lake County, Illinois, United States], Chembio HIV1/2 STAT-PAK [Chembio Diagnostic Systems Inc., Medford, New York, United States], INSTI HIV1/2 [bioLytical® Laboratories, Richmond, Canada]).^15^ Quality assurance tests, including safeguarding misclassification of HIV status, were further conducted at external laboratories. The analysis of RDS data requires adjustment for social network size and homophily (the measurement of contact between people based on characteristics) within networks. We used both RDS Analyst and STATA statistical package version 17 for the analysis of results. Chi-square tests and Student’s *t*-tests were used to compare participants’ anal sexual history characteristics (including number of lifetime sexual partners, mean age at anal sexual debut, age of first anal sex partner, exchanging money or goods for sex, whether the anal sex was receptive or insertive), condom uses with various partners during anal sex, and reasons for condomless anal sex between HIV-positive and HIV-negative MSM. The reasons assessed include being drunk or high, type of partner, fear of partner, and insertive or receptive anal sex, when the partner does not ejaculate inside. Our main outcome of interest was any CAI in the respondent’s lifetime. For continuous variables we used the mean and standard deviation, as the sample size was large enough to account for no-normality of continuous data. The significance level was kept at *P* = 0.05.

### Ethical considerations

The study was approved by the Medical Research Council of Zimbabwe, Columbia University Institutional Review board and US Centers for Disease Control and Prevention Associate Director of Science. MRCZ/A/2156. Potential participants were invited to attend an initial visit at the survey offices, during which survey staff verified coupons and screened potential participants for eligibility. If eligible and interested, survey staff obtained written informed consent from each participant in either English, Shona, or Ndebele based on participant preference.

## Results

A total of 1538 MSM were enrolled in the survey, 1511 of whom were tested for HIV and analyses were limited to this population. Table 1 presents the baseline characteristics of these participants. Most of the study participants were Black African in the age range 20 years – 40 years. More than 70% had attained a secondary level of education. The majority were never married. The unemployment rate was around 30% for both HIV-positive and HIV-negative participants.

**TABLE 1:** Baseline characteristics of men who have sex with men by HIV status.

Variable	HIV-positive (*N* = 340)		HIV-negative (*N* = 1171)		*P*
	*n*	%	*n*	%	
**Age category in years**	-	-	-	-	0.001
18–19	11	3	163	14	-
20–29	150	44	701	60	-
30–40	110	32	230	20	-
40–50	55	15	53	5	-
50+	14	4	24	2	-
**Area of residence**	-	-	-	-	0.361
Harare	149	44	546	47	-
Bulawayo	191	56	625	54	-
**Race**	-	-	-	-	0.035
Black African people	327	96	1152	98	-
White people	0	0	2	0	-
Coloured people	12	4	17	2	-
Indian people	0	0	1	0	-
Asian people	1	0	0	0	-
**Highest level of education**	-	-	-	-	0.222
None	2	1	2	0	-
Primary	23	7	55	5	-
Secondary	245	72	827	71	-
Tertiary	55	16	226	19	-
Vocational	15	4	61	5	-
**Marital status**	-	-	-	-	0.001
Single, never married	239	70	993	85	-
Married (to one woman)	12	4	63	5	-
Married (to one man)	4	1	6	1	-
Married (to more than one woman)	1	0	0	0	-
Married (to more than one man)	0	0	1	0	-
Separatedor divorced	73	21	95	8	-
Widowed	2	1	8	1	-
Cohabiting	9	3	5	0	-
**Employment status**	-	-	-	-	0.001
Self-employed	103	30	262	22	-
Employed full-time	59	17	149	13	-
Employed part-time	35	10	123	11	-
Full-time student	20	6	184	16	-
Retired	2	1	3	0	-
Unemployed	121	36	450	30	-
**Religion**	-	-	-	-	0.437
Traditional	10	3	32	3	-
Roman Catholic	75	22	25	19	-
Protestant	47	14	194	17	-
Pentecostal	83	24	338	29	-
Apostolic sect	24	7	60	5	-
Other Christian	31	9	88	8	-
Muslim	4	1	8	1	-
None	65	19	220	19	-
Other	1	0	6	1	-

Table 2 shows anal sexual history characteristics. The mean number of lifetime anal sex partners was higher among the HIV-positive MSM than those who were HIV negative. The ages of first time with any sexual or anal sexual partners were higher for the HIV-positive MSM compared to the HIV-negative. More of the HIV-positives exchanged money or goods at first-time anal sex, and the same trend holds for those who ever exchanged money or goods for sex in their lifetime. About 15% engage in both receptive and insertive anal sex, and some MSM also engaged in oral, vaginal and anal sex with women.

**TABLE 2:** Characteristics of anal sexual history.

Variable	HIV-positive (*N* = 340)				HIV-negative (*N* = 1171)				*P*
	Mean ± s.d.	*N*	*n*	%	Mean ± s.d.	*N*	*n*	%	
Number of lifetime anal sex partners	36 ± 211	-	-	-	11 ± 24	-	-	-	0.001
Age at first time with any sex partner	21 ± 7	-	-	-	20 ± 5	-	-	-	0.015
Age of first-time anal sex partner	25 ± 7	-	-	-	23 ± 6	-	-	-	0.001
Exchanged money or goods at first-time anal sex	-	340	62	18	-	1171	139	12	0.002
Ever exchanged money or goods for sex	-	340	127	37	-	1171	272	23	0.001
**Recent anal sex type**	-	-	-	-	-	-	-	-	0.001
Receptive	-	331	138	42	-	1113	307	28	-
Insertive	-	331	145	44	-	-	635	57	-
Both	-	331	48	14	-	-	171	15	-
Ever had oral sex with a woman	-	340	91	27	-	1171	409	35	0.005
Ever had vaginal sex with a woman	-	340	186	55	-	1171	726	62	0.016

s.d., standard deviation.

The prevalence of condom use or non-use with various anal sexual partners is shown in Table 3. The HIV-positive MSM had more anal sex partners in the previous 6 months compared to the HIV-negative MSM. Percentage of condomless anal sex was higher among HIV-positive MSM compared to the HIV-negative MSM. On further analysis of the frequency of condom use with anal sex partners in the last 6 months, the HIV-positive MSM had riskier behaviour. Although not statistically significant, condomless anal sex percentages with non-paying sexual partners were also higher among HIV-positive MSM compared to HIV-negative MSM. Those who were HIV positive experienced more cases of condom breaking during anal sex than the HIV-negative MSM. Those who were HIV positive had a lower percentage of ever taking pre-exposure prophylaxis (for HIV) (PrEP) than HIV-negative MSM.

**TABLE 3:** Condom use with various sexual partners in the last 6 months.

Variable	HIV-positive (*N* = 340)				HIV-negative (*N* = 1171)				*P*
	Mean ± s.d.	*N*	*n*	%	Mean ± s.d.	*N*	*n*	%	
Number of anal sex partners in the last 6 months	5 ± 11	-	-	-	3 ± 5	-	-	-	0.001
Did not use a condom with a regular anal sex partner	-	340	126	37	-	1171	354	30	0.017
**Frequency of condom use with anal sex partner in last 6 months**	-	-	-	-	-	-	-	-	0.023
Always	-	335	141	42	-	1138	529	46	-
Most of the time	-	335	34	10	-	1138	171	15	-
Sometimes	-	335	53	16	-	1138	135	12	-
Rarely	-	335	35	10	-	1138	100	9	-
Never	-	335	72	21	-	1138	203	18	-
Number of anal non-paying sex partners in the last 6 months	2 ± 6	-	-	-	1 ± 3	-	-	-	0.001
Did not use a condom with a non-paying anal sex partner	-	170	41	24	-	500	97	19	0.189
**Frequency of condom use with anal non-paying sex partner in last 6 months**	-	-	-	-	-	-	-	-	0.408
Always	-	170	96	56	-	500	306	61	-
Most of the time	-	170	16	9	-	500	56	11	-
Sometimes	-	170	28	16	-	500	57	4	-
Rarely	-	170	11	6	-	500	35	7	-
Never	-	170	19	11	-	500	46	9	-
Cases of a condom breaking during anal sex	-	340	106	31	-	1168	272	23	0.003
Unable to ask the main male sexual partner to use a condom	-	340	39	11	-	1168	115	10	0.376
Ever taken PrEP	-	95	21	22	-	534	167	31	0.072

PrEP, Pre-exposure prophylaxis (for HIV); s.d., standard deviation.

Table 4 shows the reasons for CAI. Misconceptions and reasons for failure to use condoms were higher among HIV-positive MSM than the HIV-negative MSM, and these were: drunkenness, partner fear, partner being regular, trusting unregular partner, coitus interuption, and being offered more money.

**TABLE 4:** Reasons for condomless anal sex.

Variable	HIV-positive *N* = 340			HIV-negative *N* = 1171			*P*
	*N*	*n*	%	*N*	*n*	%	
Did not use a condom when drunk or high	339	119	35	1171	303	26	0.001
Did not use condoms because they are afraid to ask their partner	339	45	13	1171	104	9	0.017
Did not use condoms because the partner is regular	339	198	58	1171	623	53	0.090
Did not use condoms because of trusting an unregular partner	338	35	10	1171	72	6	0.008
Did not use a condom because he is a top partner (insertive)	337	39	12	1167	87	7	0.016
Did not use a condom because he is a bottom partner (receptive)	336	30	9	1158	75	6	0.122
Did not use a condom when there is agreement that partner does not ejaculate inside	338	22	7	1169	49	4	0.077
Did not use a condom when offered or offer more money	338	34	10	1171	64	6	0.003
Did not use condoms for other reasons	340	20	6	1171	49	4	0.187
**Condom use percentages by type of sex**	-	-	-	-	-	-	0.001
When his penis is in me	333	80	24	1157	186	61	-
When my penis is in him/her	333	112	34	1157	495	43	-
Equally likely	333	141	42	1157	476	41	-

Other reasons for CAI collected qualitatively include knowledge of partner status, unpreparedness, condom unavailability, being excited or carried away, disliking condoms, being in prison, having the perception that anal sex is safe, having an attractive partner, being embarrassed to get a condom, and condoms causing a rash.

## Discussion

We found a high prevalence of CAI of at least 30% among HIV-positive and HIV-negative MSM. We also note that self-reported sexual behaviour may be subject to error and bias, thus underreporting of CAI is possible and may have led to underestimation of prevalence. HIV prevalence was high at 22.5%. In the context of the high HIV prevalence of 11.6%^16^ in the general population and 23.4%^14^ among MSM, the levels of CAI observed in this study are worrying.

High levels of CAI might explain the elevated HIV prevalence in this key population.^5^ Our results pointed to various preventable factors that led to CAI, including the perception that anal sex is safe, trusting an irregular or regular partner, partner fear, having a partner who does not ejaculate inside, unpreparedness, condom unavailability, being embarrassed to get a condom, being offered or offering more money, having a knowledge of partner status, drunkenness, being excited or carried away, having an attractive partner, disliking condoms, condoms causing a rash, and being in prison. We also noted that the participants reported multiple sexual partners in the previous 6 months, which would lead to widespread HIV transmission as a result of condomless sexual acts with these partners. Another finding was that some of the MSM also engage in oral, vaginal and anal sex with women. Our analysis shows a concerning finding that some MSM believe CAI is acceptable under some circumstances, including situations when they are not prepared, or when there is a false perception that anal sex is safe depending on the partner. These behaviours are likely to contribute to further HIV transmission and high prevalence among MSM. However, we note that at least a fifth of the participants had ever taken PrEP, which may have been a confounder for CAI. A study has shown that MSM PrEP users may tend to practise condomless anal sex because they perceive that PrEP use decreases their risk of HIV infection.^9^ However, with PrEP and Undetectable = Untransmittable not yet actualising their full potential worldwide, there is concern that overemphasis on these innovations may cause HIV-affected people to ignore other concerns related to condomless sex, including other STIs.^17^ As PrEP becomes increasingly available and utilised in Zimbabwe, future research should take it into account when assessing sexual risk behaviour and modes of HIV transmission.

These findings suggest that the high prevalence of CAI among MSM in Zimbabwe is a complex issue affected by a range of factors.

Another interesting finding is the various percentages of CAI depended on sexual positioning, that is whether or not one is insertive or receptive. Risks for acquiring or transmitting HIV and STIs via condomless anal sex vary according to sexual positioning. Specifically, men who participate in receptive anal intercourse were more likely to acquire HIV and rectal STIs compared to men who only participate in insertive anal intercourse.^18^ A greater understanding of the dynamics underlying sexual positioning practices and the ways these dynamics may contribute to HIV and other STIs is needed.^18^

MSM are an important key population for HIV prevention and treatment in SSA and globally. Interventions to increase condom use and other sexual risk reduction strategies among MSM will need to address the situational context in which these men engage in sex and develop strategies that are adaptable to their environments. These efforts should not only focus on enhancing condom availability and access but also on tailored education to address misconceptions about HIV transmission risks and the benefits of condom use. Furthermore, interventions that tackle socio-economic factors, such as financial incentives for safe sex, may also be beneficial in this context. Importantly, this underscores the need for interventions to be multidimensional, addressing the intertwined nature of these risk factors. In fact, tackling accessibility issues without addressing the socio-behavioural and perceptual barriers may not effectively reduce CAI rates. As such, an integrated and holistic approach is necessary to mitigate the high levels of CAI and the subsequent elevated HIV prevalence in the MSM population in Zimbabwe. Outreach programmes, for example, have frequently been identified as a powerful strategy to engage these populations with vital health services.^19^

## Conclusion

Overall, our findings indicate that there is potential for further HIV transmission among MSM in Zimbabwe, and an urgent need for increased HIV prevention efforts among this group in relation to optimising condom use. The main limitation is that our study was cross-sectional and based on behaviours reported in a face-to-face interview, and may be prone to social desirability responses. However, to our knowledge, this is the first large BBS that generated valuable information useful for analysing condomless anal sex among MSM in Zimbabwe.
